# Isolation of AMP-activated protein kinase (AMPK) alleles required for neuronal maintenance in *Drosophila melanogaster*

**DOI:** 10.1242/bio.20136775

**Published:** 2013-10-22

**Authors:** Lance L. Swick, Nevzat Kazgan, Rob U. Onyenwoke, Jay E. Brenman

**Affiliations:** Department of Cell Biology and Physiology, and Neuroscience Center, University of North Carolina–Chapel Hill School of Medicine, Chapel Hill, NC 27599, USA

**Keywords:** AMPK, *Drosophila*, Energy homeostasis, Kinase, Neurons, AMP-activated protein kinase

## Abstract

The maintenance of energetic homeostasis in the face of limited available nutrients is a complex problem faced by all organisms. One important mechanism to maintain energetic homeostasis involves the activation of the energy sensor AMP-activated protein kinase (AMPK). AMPK is a cell-autonomous energy sensor that is highly sensitive to and regulated by the ATP to ADP and ATP to AMP ratios. However, the genetic analysis of AMPK signaling in vertebrates has been complicated by the existence of multiple redundant AMPK subunits. Here, we describe the identification of mutations in the single *Drosophila melanogaster* AMPK catalytic subunit (AMPKα) and their implications for neural maintenance and integrity. This article provides a citation replacement for previously published *ampkα* alleles, transgenes and neuronal phenotypes, which remain accurate; however, they were used in a previously published study that has subsequently been retracted (Mirouse et al., 2013).

## Introduction

AMP-activated protein kinase (AMPK) acts as a cellular energy sensor and is activated by ADP and AMP, which accumulate when ATP levels are low (Braco et al., 2012; Johnson et al., 2010; Kahn et al., 2005). AMPK then mediates the cellular response to energetic stress by activating energy-producing activities, while inhibiting energy-consuming ones, such as protein translation and cell growth/division. AMPK is a heterotrimeric protein with a 63-kDa catalytic α subunit and two regulatory β and γ subunits (38 and 36 kDa, respectively), each of which is encoded by distinct genes (α1, α2; β1, β2; γ1, γ2, γ3 in mammals; Davies et al., 1994; Gao et al., 1996; Mitchelhill et al., 1994; Nielsen et al., 2003; Stapleton et al., 1996). AMPK is also implicated in a number of signaling pathways (Hardie, 2004; Hardie, 2007; Shaw, 2009).

AMPK can be activated upstream by the tumor suppressor liver kinase B1 (LKB1; Amin et al., 2009; Shaw et al., 2004) and Ca^2+^/calmodulin-dependent kinase kinase β (Hawley et al., 2005; Hurley et al., 2005). AMPK signaling acts downstream to inhibit protein and lipid synthesis, for example, activating elongation factor-2 kinase, which causes inhibition of the elongation step of translation (Horman et al., 2002; Winder et al., 1997); inhibiting the target-of-rapamycin (TOR) pathway, which stimulates the initiation step of protein synthesis by the phosphorylation of multiple targets (Proud, 2004); and phosphorylating and inhibiting the fatty acid synthesis and rate-limiting enzyme ACC, which subsequently lowers malonyl-CoA levels and increases fatty acid uptake into mitochondria (Hardie and Hawley, 2001; Merrill et al., 1997; Winder et al., 1997).

We have further investigated the role of AMPK *in vivo* and show here that AMPK is required to maintain cellular integrity in neurons within *Drosophila melanogaster*.

## Results and Discussion

**Fig. 1.**
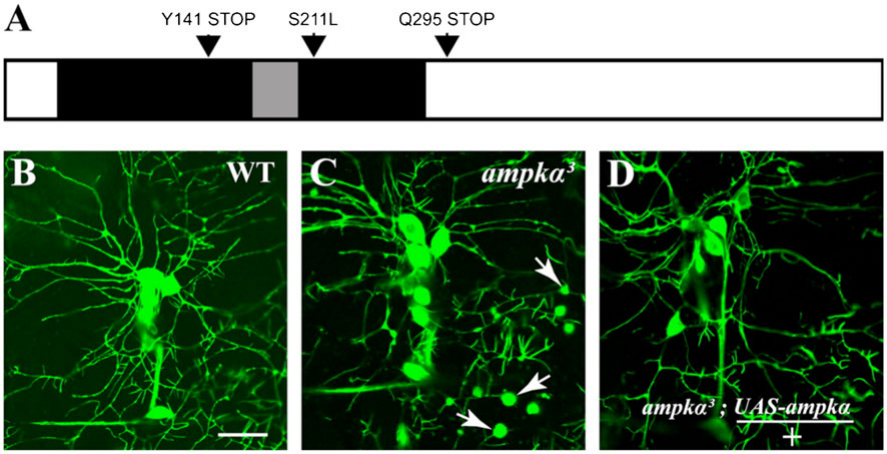
Identification of mutations within the single *Drosophila melanogaster ampkα* gene. (A) Schematic domain representation of AMPKα and corresponding genetic lesions in mutants. The serine/threonine kinase domain (black, aa 39–280) and T-Loop (gray, aa 167–194) are shown with the sites of mutations, S211L, Q295STOP, and Y141STOP, for *ampkα^1^*, *ampkα^2^*, and *ampkα^3^*, respectively. (B) Representative image of wild-type da neurons expressing an *Actin::GFP* fusion transgene in a second instar larva. (C) *ampkα* mutants display enlarged plasma membrane domains (arrows) in sensory neuron dendrites, but not axons. (D) A wild-type *ampkα* transgene expressed autonomously within da neurons completely rescues the dendrite phenotype. (B–D) Background genotypes are *w; Gal4109(2)80, UAS-actin::GFP*. Anterior toward the left and dorsal toward the top. Bars, 20 µm.

AMPK contains three protein subunits, α, β, and γ, which form a heterotrimer. The α subunit (AMPKα) encodes for a highly conserved serine/threonine kinase, whereas the other subunits are regulatory. From a *Drosophila melanogaster* forward genetic screen for mutants affecting larval neuronal dendrite development and maintenance (Medina et al., 2006), we identified several lethal mutations in *AMPKα*. The ethyl methane sulfonate (EMS) mutants, *ampkα^1^* and *ampkα^2^*, contain a single amino acid change (S211L, completely conserved) and a premature stop codon (Q295 STOP), respectively, whereas *ampkα^3^* has a 16-bp deletion creating a stop codon (Y141 STOP; Fig. 1A). All *ampkα* mutants, whether homozygous or in trans with a deletion covering the locus, displayed a completely penetrant and nearly identical phenotype, with significantly enlarged plasma membrane domains in dendrites, but not in axonal compartments (Fig. 1C; unpublished data). In addition, *ampkα^1^* and *ampkα^3^* could be rescued to viability with either a chromosomal duplication carrying a wild-type *ampkα* gene, a wild-type AMPKα transgene, or a transgene that is tagged with the red fluorescent protein mCherry (Fig. 1D; see Materials and Methods). The requirement for *ampkα* is cell autonomous because transgene expression within only neurons rescues the phenotype (Fig. 1D). Therefore, these mutations represent genetic disruptions of the single AMPKα catalytic subunit in the *D. melanogaster* genome. (These data and these alleles were previously published as a part of a retracted study (Mirouse et al., 2013) but accurately allow the genetic analysis of AMPK function *in vivo*.)

The recovery of null mutations in *ampkα* has allowed for *in vivo* analysis of AMPK function in a multicellular organism, which has revealed an unexpected role for the kinase in the maintenance of cell integrity. This implies that at least one of the pathways that normally maintain cell integrity cannot function without AMPK activity. These results are in agreement with previous studies that have investigated the role of both AMPKβ and AMPKγ in neural maintenance and neurodegeneration in *Drosophila*, and found that both of these AMPK regulatory subunits are also essential to the maintenance of neural integrity (Andersen et al., 2012; Spasić et al., 2008; Tschäpe et al., 2002).

## Materials and Methods

### Mutant AMPK*α* allele isolation and characterization

An ethyl methane sulfonate mutagenesis screen on the X chromosome was performed as previously described (Medina et al., 2006). Early second instar larvae were visually screened for dendritic defects using fluorescent microscopy. The *ampkα* mutants, lethal at late second/third instar stages, were mapped to ∼150 kb on the X chromosome using a molecularly defined deficiency (*Df[1]Exel6227*), an undefined deficiency (*Df[1]AD11*), and a duplication of the Y chromosome (*Dp[1;Y]/Df[1]svr*). Predicted coding regions for genes in the region were sequenced using PCR amplicons made from mutant genomic DNA, and one gene (*AMPKα*; *CG3051*; NM_057965) was discovered that had significant mutations in all three alleles. Alleles have been contributed to the Bloomington Stock Center.

### Construction of *AMPKα* transgenes

The wild-type *AMPKα* transgene was cloned into the *pUAST* vector (Brand and Perrimon, 1993) as an *EcoR*I–*Bg*lII fragment of an EST, corresponding to an *AMPKα-RA* transcript (http://flybase.org). The mCherry-AMPKα fusion protein was made using a mCherry construct (provided by R. Tsien, University of California, San Diego, San Diego, CA) at the N terminus fused in-frame to *AMPKα* into the *pUAST* vector. The *UAS-mCherry-AMPKα* transgene rescues viability and fertility when expressed by *Ubiquitin-Gal4* in either *ampkα^1^* or *ampkα^3^* mutants. The phosphomimetic-activated form of AMPKα (AMPKα T184D) was made by PCR-based, site-directed mutagenesis converting base C549 to G549. The transgenes were introduced into a *w^1118^* stock by *P* element-mediated transformation. Transgenes have been contributed to the Bloomington Stock Center.

### Neuron visualization technique

Larvae were covered in a glycerol solution at 22°C and gently covered with a coverslip (22×50 mm; Fisher Scientific) to restrict movement, but not cause bursting of the body wall. Images were quickly taken using a Pan-Neofluar 40×/1.3 NA oil immersion lens with a 2-µm optical slice and LSM Imaging software (Carl Zeiss MicroImaging, Inc.). Images were resized and cropped with Photoshop (Adobe), and imported into Illustrator (Adobe) for labels and arrangement.
